# Complete Resolution of Paraneoplastic Membranous Nephropathy Following Curative Therapy of Triple-Negative Breast Cancer

**DOI:** 10.7759/cureus.18125

**Published:** 2021-09-20

**Authors:** Muhammad B Khan, Amandeep Kaur, Asad Ali, Avezbakiyev Boris, Samuel Spitalewitz

**Affiliations:** 1 Nephrology, Brookdale University Hospital Medical Center, Brooklyn, USA; 2 Medicine, Brookdale University Hospital Medical Center, Brooklyn, USA; 3 Hematology/Oncology, Brookdale University Hospital Medical Center, Brooklyn, USA

**Keywords:** triple-negative carcinoma of the breast, membranous nephropathy, paraneoplastic syndrome, breast cancer, phospholipase a2 receptor

## Abstract

A paraneoplastic syndrome, which includes glomerulopathy, is a manifestation of malignancy unexplained by direct tumor burden. Membranous nephropathy (MN) may be associated with malignancies that are primarily solid tumors of the lung, prostate and gastrointestinal tract. It is rarely associated with breast cancer. To our knowledge, we herein report the first case of MN associated with triple-negative carcinoma of the breast. The patient initially presented with MN as a paraneoplastic nephrotic syndrome. Treatment resulting in a complete pathological response of the breast cancer also resolved the MN. Neither has recurred after a 48-month follow-up. The patient exhibited autoantibodies against phospholipase A2 receptor and was also antinuclear antibody (ANA) and anti-Smith (anti-Sm) antibody positive. These results suggest that the neoplasm evoked an autoimmune response, which resolved with treatment. ANA and anti-SM positivity closely correlated with the neoplasm activity supporting this hypothesis.

## Introduction

Paraneoplastic syndrome is the manifestation of a malignancy that is not explained by direct tumor burden, the extent of invasion or a metastatic process of the disease [1]. Paraneoplastic glomerulopathies were first suggested in 1966 by Lee et al. [2]. Subsequently, systematic reviews and meta-analyses of observational studies in 2014 reported that 10% of patients with membranous nephropathy (MN) had an associated malignancy [3]. The majority of these malignancies were solid tumors of the lung, prostate and gastrointestinal tract.

Breast cancer is the most common malignancy in women, yet paraneoplastic glomerular diseases are rarely linked to breast cancer [3,4]. In a review by Bacchetta et al., there were only five reported cases of breast cancer among 159 patients with MN, and none were triple-negative (estrogen receptor, progesterone receptor, and epidermal growth factor receptor 2 [HER-2]) [5]. To our knowledge we herein report the first case of triple-negative breast cancer presenting with MN as a paraneoplastic manifestation which resolved with successful treatment of the carcinoma.

## Case presentation

The patient is a 46-year-old Hispanic woman who initially presented with bilateral leg swelling and abdominal distention that progressed over the prior three months, associated with a 20-pound weight loss, fatigue and loss of appetite. One month before presentation, she was diagnosed with triple-negative invasive ductal carcinoma of the breast. The patient reported no abdominal pain, chest pain, shortness of breath, bone pain, arthritis, arthralgia, malar rash, recent polyuria or polydipsia. No family history of malignancies or kidney diseases was reported. There was no history of intravenous drug use or smoking. Blood pressure was 105/72 mmHg, heart rate was 85 beats per minute, respiratory rate was 18 breaths per minute and temperature 36.9°C. The rest of the physical examination was significant only for the presence of anasarca and a 2.5-cm non-tender mass in the upper outer quadrant of the left breast with palpable left axillary lymph nodes. Initial laboratory findings included the following: serum Na 129 meq/L, albumin 1.8 g/dL, total cholesterol 315 mg/dL, HDL 126 mg/dL, LDL 215 mg/dL and triglycerides 283 mg/dL. Serum creatinine (Cr) was normal at 0.9 mg/dL. Urinalysis revealed 3+ protein with 0-5 RBCs/hpf; a urine protein/Cr ratio of 16.4 g/g Cr and a urinary protein excretion of 14 g/24 hr. A CT scan of the chest, abdomen and pelvis disclosed ascites, a mass in the lateral left breast, left axillary lymphadenopathy, and several bilateral lower lobe segmental pulmonary emboli. The patient was treated with low molecular weight heparin for bilateral pulmonary embolism. A mammogram examination revealed a 2.5-cm mass in the upper outer quadrant of the left breast. The pathology report on the subsequent breast tissue biopsy demonstrated invasive ductal carcinoma with medullary carcinoma features, histological grade 3 (overall score 8: tubular differentiation 3, nuclear pleomorphism 3, and mitotic rate 2; estrogen receptor-negative [0%]; progesterone receptor-negative [0%]; HER-2 not overexpressed).

The biopsy of the left axillary lymph node showed metastatic adenocarcinoma. A bone scan reported no evidence of bone metastases. Although anasarca was present, liver function tests were normal and an echocardiogram showed normal left ventricular function. Serology tested negative for HIV, hepatitis B, hepatitis C and antithrombin III. The antinuclear antibody (ANA) test was positive in a speckled pattern at a titer of 1:640, anti-Smith (anti-Sm) antibody-positive at a titer of 7.5 AI, and anti-SM/RNP positive at a titer >8. Double-stranded DNA was not reported. The levels of serum C3 and C4 were in the normal range, 124 mg/dL and 32 mg/dL, respectively.

**Figure 1:**
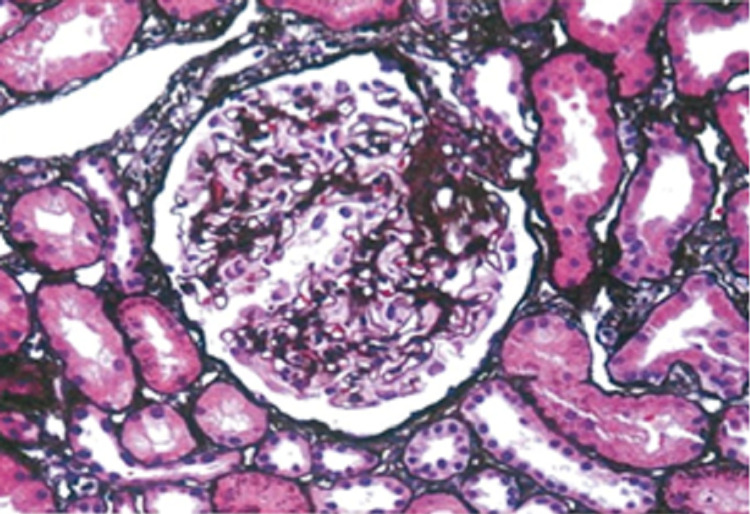
Light microscopy Thickened glomerular capillary walls with open lumens, minimal focal interstitial fibrosis and no infiltrate of inflammatory cells (Jones silver stain; 400x).

**Figure 2:**
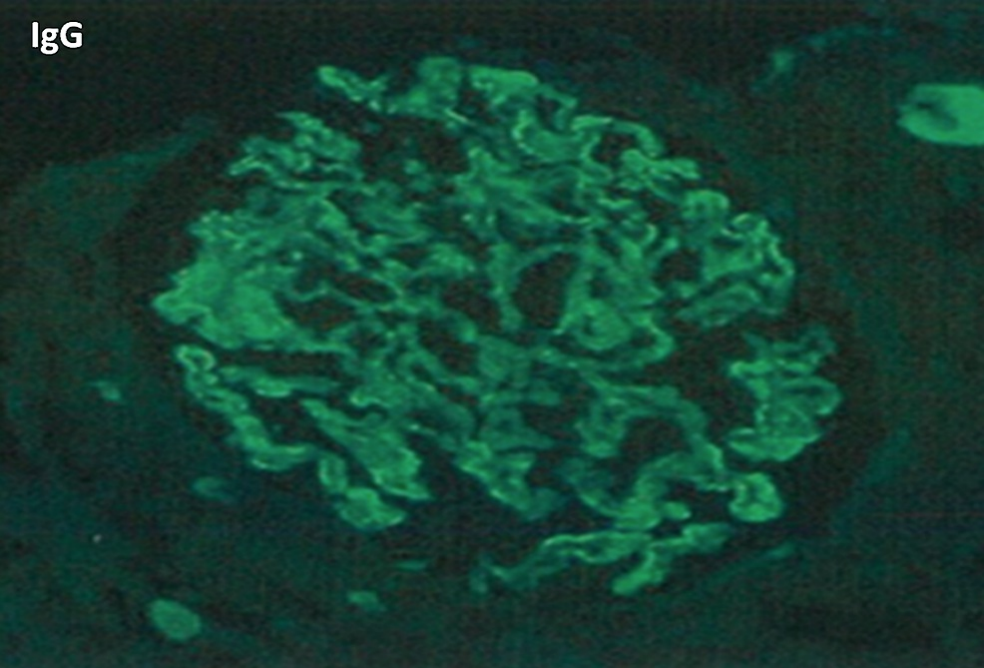
Direct immunofluorescence stain Diffuse granular staining in capillary walls for IgG (3+).

The patient underwent a renal biopsy. On light microscopy, the glomerular capillary walls were thickened with open lumens, accompanied by minimal interstitial fibrosis and no infiltration with inflammatory cells (Figure 1). Direct immunofluorescence showed diffuse granular staining in capillary walls for IgG (3+), IgA (1+), C3 (2+), C1q (1+), and kappa (2-3+) and lambda (3+) light chains with C1q weakly positive along GBM (Figure 2). Subclasses of IgG by immunofluorescence were not performed.

**Figure 3:**
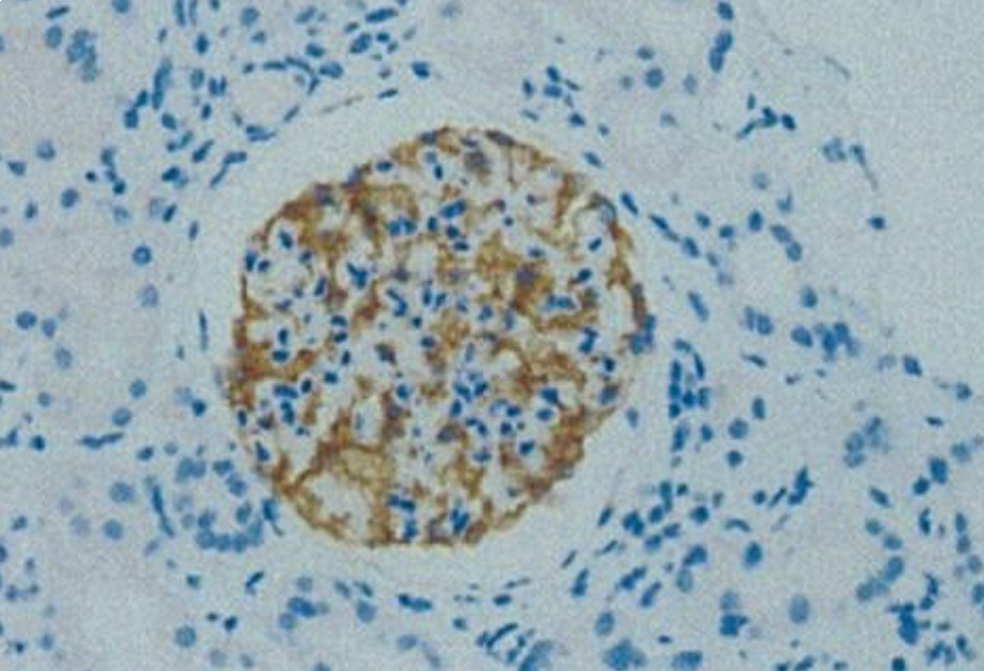
Immunohistochemistry staining Positive staining for PLA2R along the glomerular basement membrane.

**Figure 4:**
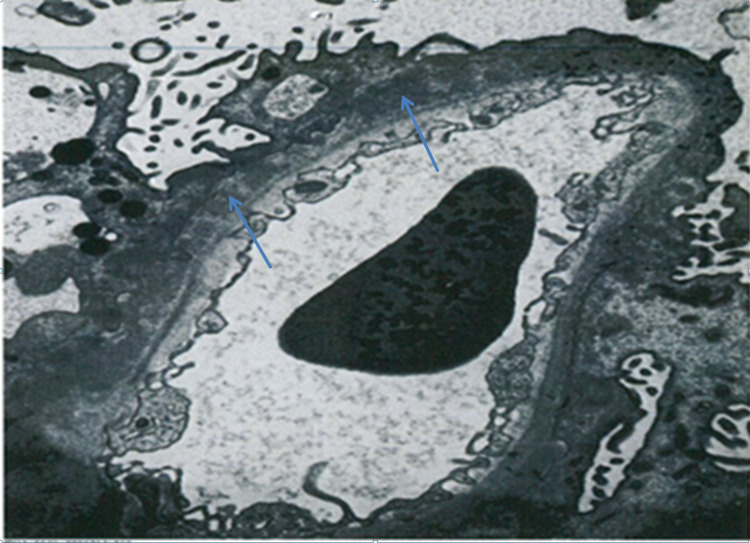
Electron microscopy Immune type electron-dense deposits in subepithelial locations; the deposits were separated and partially surrounded by irregular projections of basement membrane-like material. Overlying epithelial cells showed marked effacement of the foot process.  Mesangial or subendothelial deposits and tubuloreticular inclusions were not seen. No immune type electron-dense deposits in the tubular basement membrane, interstitium or peritubular capillaries were seen.

On immunohistochemistry staining the glomerular capillary walls stained positive for anti-phospholipase A2 receptor (PLA2R) (Figure 3). On electron microscopy, numerous subepithelial electron-dense deposits were seen, accompanied by diffuse effacement of the foot processes. Mesangial or subendothelial deposits and tubuloreticular inclusions were not seen (Figure 4). These findings were consistent with MN stage III, featuring positive staining for anti-PLA2R and full-house immunoglobulins.

**Figure 5:**
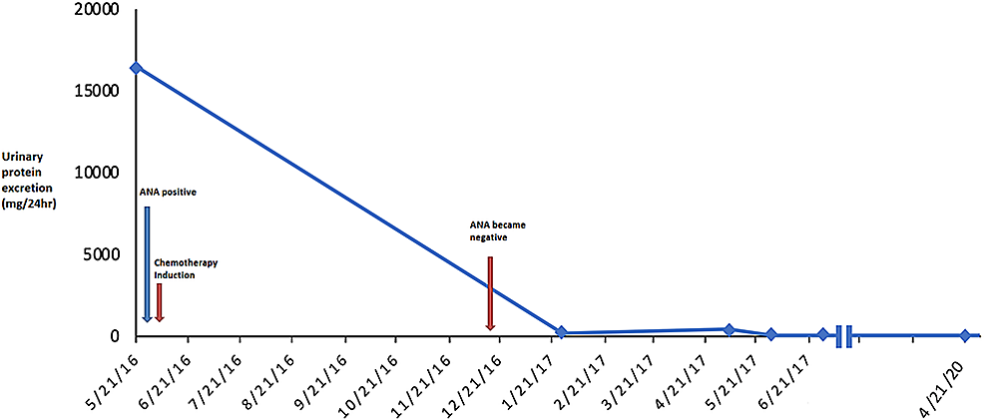
Clinical course of the disease Marked decreases in urinary protein excretion corresponding to changes in the antinuclear antibodies (ANA) test in response to chemotherapy.

The patient received neoadjuvant chemotherapy consisting of doxorubicin and cyclophosphamide for four cycles followed by a taxane (initially paclitaxel and then switched to docetaxel because of allergic reactions) for four cycles and four cycles of dexamethasone. In response to the chemotherapy, the breast mass significantly decreased in size. The nephrotic syndrome resolved completely. The 24-hour urinary protein excretion estimated by urinary protein/Cr ratio decreased from 16,433 mg/24hr to 75 mg/24 hr and the serum albumin level and lipid profile normalized. The patient underwent a radical mastectomy of the left breast with axillary lymph node dissection. Pathological examination of the surgical specimen revealed no evidence of residual neoplasm, which is considered a pathological complete response. The patient completed radiation therapy to the breast. Surveillance mammogram examinations did not show recurrence of malignancy after six-month, 12-month, 24 and 48-month follow-up. There has been no recurrence of proteinuria 48 months after successful treatment of the primary tumor. The clinical course is depicted in Figure 5.

## Discussion

The term paraneoplastic syndrome refers to clinical manifestations not directly related to tumor burden, invasion or metastasis, and should be suspected when there is no apparent alternative etiology available to explain the clinical findings [4]. There is a temporal relationship between the syndrome and the malignancy and the resolution of the paraneoplastic syndrome occurs with successful treatment of the primary tumor [1,6]. The reappearance of the paraneoplastic syndrome usually occurs with recurrence of the tumor [1,6-8]. Our patient developed symptoms and signs of the nephrotic syndrome in the weeks prior to being diagnosed with breast cancer. The patient’s breast cancer responded well to the chemotherapy regimen as evidenced by complete pathological response demonstrated on the mastectomy specimen. During the course of the chemotherapy, the proteinuria resolved completely and remained negative through a 48-month follow-up.

Eagen reported in 1977 that both complete remission of MN and malignancy rarely occur together since the malignancy is often incurable [9]. In contrast, our patient achieved both remissions of her malignancy and MN. In paraneoplastic glomerulopathies, the glomerulopathy and the malignancy may not recur at the same time. The two conditions may be independent of one another [8,10,11]. In our patient, however, neither has recurred after 48 months of follow-up.

MN is an autoimmune disorder typically associated with nephrotic syndrome. The histological alterations are characterized by subepithelial deposits of immunoglobulins and thickening of the glomerular capillary wall. In primary MN (PMN), the etiology is often unknown, whereas the etiology is recognizable in secondary MN. In PMN, the glomerular lesions are caused by deposition of autoantibodies against a podocyte membrane protein. The pathogenesis of PMN was first elucidated in the rat model of Heymann nephritis [12]. In this model, deposition of the subepithelial IgG results from in situ immune complex formation, involving antibodies against podocyte antigens, followed by local complement activation to form the membrane attack complex, C5b-9 [13-15]. The resulting injuries to the glomerular basement membrane lead to proteinuria, which is preceded by the initiation of antibody response by weeks or months. In the seminal work by Beck et al. [13], patients with PMN have IgG4 autoantibodies against PLA2R, a podocyte antigen, both in the circulation and in GBM immune deposits. In various reports, anti-PLA2R antibodies have been identified in 52%-78% of patients with PMN [16]. A second IgG4 antibody against another podocyte antigen, thrombospondin type 1 domain-containing 7A (THSD7A), was later found in 2%-5% of patients with PMN [17]. The co-localization of PLA2R or THSD7A with the IgG in GBM deposits strongly implies the pathogenic role of these autoantibodies. Approximately 10% of patients with PMN test negative for both antibodies; thus, other autoantibodies to podocyte antigens are likely to be identified. Specific immunogenetic risk alleles appear to account for some individuals more prone to the development of MN [18]. 

A distinction of PMN from secondary MN is necessary to determine therapy. In addition to supportive care to reduce proteinuria and protection of renal function, patients with PMN are treated with immunosuppressive agents once the decision to treat is made based upon the likelihood of disease progression. Accepted immunosuppression regimens include glucocorticoids combined with cyclophosphamide, calcineurin inhibitors alone or in combination with glucocorticoids, and B-cell depletion [17]. In contrast, therapy of secondary MN targets the disease etiology associated with MN. For example, the nephrotic syndrome due to MN associated with hepatitis B may be brought into remission once hepatitis B is eradicated with antiviral therapy. Similarly, MN secondary to medications resolves after discontinuation of the offending drugs. MN is most commonly idiopathic but secondary causes such as infection, systemic lupus erythematosus (SLE), and malignancy may be detected in 20%-30% of adults. The prevalence of MN related to malignancy is 6%-22% [5]. Most of these cases had tumors originating from the gastrointestinal tract, lung or prostate [4]. To our knowledge, only five cases of breast cancer associated with MN have been reported in the literature with none of them being triple-negative breast cancer [5].

In our patient, the histopathological findings and corresponding clinical course pose three differential possibilities to be considered: (1) PMN occurring coincidentally with breast carcinoma; (2) secondary lupus MN occurring coincidentally with breast cancer; and (3) breast carcinoma precipitating autoimmunity and resulting in the development of the autoimmune disorder of MN. In our case, positive immunohistochemistry staining for PLA2R suggests the presence of the primary form of MN. However, the nephrotic syndrome completely resolved, closely following curative therapy of breast cancer. Although PMN may spontaneously resolve, the clinical course and response to therapy argue for a secondary form of MN associated with the breast neoplasm rather than remission of a primary form of MN. Alternatively, the positive serology test for ANA raises the possibility that SLE is the causative etiology for secondary MN. There are, however, several clinical and laboratory observations that make this consideration improbable and more likely the breast carcinoma precipitated autoimmunity. First, the patient did not present with clinical manifestations of SLE. Second, the appearance of a positive test for ANA occurred when the breast cancer was diagnosed. Third, the ANA test became negative after eradication of the breast cancer. Finally, patients with secondary MN associated with SLE typically maintain lifelong ANA positivity. In our patient, the timing of ANA serology changes coincides with the development of breast cancer and its eradication, suggesting that the neoplasm elicited an autoimmunity reaction. In addition, serum C3 and C4 were normal and histologic staining for C1q was weak. No mesangial, sub-endothelial deposits or tubuloreticular inclusions were seen. Additionally, there were no immune-type electron-dense deposits in the tubular basement membranes. At presentation, anti-Sm antibodies, which are fairly specific but certainly not diagnostic for lupus, were positive (greater than 7.5) but subsequently were normal, less than 1.0, last documented in 2017 correlating with successful cancer therapy. Similarly anti-SM/RNP, an extremely rare finding in PMN [19], normalized with successful therapy of the neoplasm.

There are autoimmune disorders that can arise from neoplasm as paraneoplastic syndrome. Lambert-Eaton myasthenic syndrome seen in patients with small-cell lung cancer and other neoplasms is an example. In this syndrome, an autoantibody-mediated attack of voltage-gated calcium channels disrupts presynaptic neurotransmitter release, resulting in a loss of function of motor nerve terminals and muscle weakness. In our patient, it is conceivable that the breast cancer activated an autoimmune process to prompt a subset of B-cells to produce autoantibodies against PLA2R and a positive ANA. Similarly, it should be noted that the presence of anti-PLA2R antibodies is not uncommon in patients with hepatitis B-associated MN. It has been hypothesized that immune perturbations by the viral infection may trigger autoantibody reactions to various epitopes of PLA2R via epitope spreading. Epitope spreading is a phenomenon wherein new epitopes within an antigen molecule previously nonrecognizable become recognized by T or B cells and is described in many autoimmune diseases. In cancer-associated MN, immune challenge by cancer might provoke the production of autoantibodies in a similar manner. In a recent report, circulating anti-PLA2R antibodies are detected in 41% of patients with cancer-associated MN [20]. This high prevalence of antibodies against PLA2R in cancer-associated MN would support a plausible causality link between cancer and autoimmunity.

Our case report has several limitations. One of the effective treatments of PMN is the Ponticelli regimen, which includes alkylating agents, either chlorambucil or cyclophosphamide. We cannot be totally secure that the remission of the nephrotic syndrome resulted from the successful treatment of the carcinoma rather than the use of cyclophosphamide. However, our patient received 4 g of cyclophosphamide (1 g every other week for four cycles) which is 25% of the dose that would have been administered if she were treated with the Ponticelli protocol. In addition, only 50%-60% of patients treated with the Ponticelli protocol achieve remission at one year [21]. Yet, on a far lower dose of cyclophosphamide, she achieved complete remission of the nephrotic syndrome by eight months or less. This response is, therefore, more compatible with successful treatment of her breast cancer and supports the possibility of paraneoplastic MN precipitated by breast cancer.

We did not identify the IgG isotype in the GBM immune deposits. Most cases of PMN display isotypic IgG4 whereas IgG1 and IgG3 are more likely the isotypes for secondary MN associated with cancer. Although the renal biopsy specimen demonstrated anti-PLA2R deposits by immunohistochemistry staining, we did not determine the dynamic presence of anti-PLA2R autoantibodies in the circulation. Serial determinations of anti-PLA2R serology were not performed when MN and breast cancer developed and when the nephrotic syndrome resolved with cancer eradication. Due to insurance and laboratory constraints, anti-phospholipase A2 could not be measured serially. Such demonstration of the appearance and disappearance of anti-PLA2R serology during the clinical course would have strengthened the possibility of autoimmunity induced by the breast cancer. Despite these limitations, our case emphasizes a critical clinical decision. Facing the decision to treat breast cancer or MN, we chose to eradicate the cancer for practical considerations. Consequent to adopting this decision, the nephrotic syndrome was resolved. This clinical response is consistent with our proposal that MN could occur as a form of paraneoplastic syndrome in patients with MN associated with breast cancer.

## Conclusions

Paraneoplastic syndrome is the manifestation of a malignancy that is not explained by direct tumor burden, the extent of invasion or a metastatic process of the disease. Paraneoplastic glomerulonephritis is a rare secondary cause of glomerulonephritis and a complication of cancer. Recognition of paraneoplastic glomerulonephritis and subsequent detection of an undiagnosed malignancy could be life-saving. The majority of these malignancies were solid tumors of the lung, prostate and gastrointestinal tract. Paraneoplastic glomerular diseases are rarely linked to breast cancer even though it is the most common malignancy in women. There are only a few reported cases of breast cancer-associated MN in the literature, and none were reportedly triple-negative (estrogen receptor, progesterone receptor, and epidermal growth factor receptor 2 (HER-2). We herein report the first case of triple-negative breast cancer presenting with MN as a paraneoplastic manifestation resulting in complete resolution of paraneoplastic MN following curative therapy of triple-negative breast cancer. Therefore, efforts to successfully treat the carcinoma are most important rather than therapy of the glomerulopathy per se.
